# Distinguishing Killian–Jamieson diverticulum from Zenker’s diverticulum

**DOI:** 10.1186/s40792-023-01599-7

**Published:** 2023-02-10

**Authors:** Yuki Watanabe, Yusuke Taniyama, Ken Koseki, Hirotaka Ishida, Yohei Ozawa, Hiroshi Okamoto, Chiaki Sato, Michiaki Unno, Takashi Kamei

**Affiliations:** Department of Surgery, Tohoku University Hospital, 1-1, Seiryo-Tyo, Aoba-Ku, Sendai, Miyagi 980-8574 Japan

**Keywords:** Killian–Jamieson diverticulum, Pharyngoesophageal diverticulum, Cricopharyngeal muscle

## Abstract

**Background:**

Killian–Jamieson diverticulum, which is a relatively rare pharyngoesophageal diverticulum, is difficult to distinguish from Zenker’s diverticulum. Because major points of the relevant surgical procedures for these two entities differ, it is important to make an accurate diagnosis. We herein report a case of Killian–Jamieson diverticulum initially diagnosed as Zenker’s diverticulum.

**Case presentation:**

A 56-year-old man complaining of discomfort during swallowing was diagnosed with pharyngoesophageal diverticulum. He was initially diagnosed with Zenker’s diverticulum before surgery, but the diverticulum actually arose from the left side of the esophageal wall, at the level of the cricoid cartilage and below the cricopharyngeal muscle. We therefore ultimately diagnosed this case as Killian–Jamieson diverticulum during surgery, and were able to preserve the muscle above the diverticulum, which would normally have to be cut when treating a case of Zenker’s diverticulum.

**Conclusion:**

To make an accurate diagnosis, clinical and surgical findings are important to consider, including the location of the diverticulum and the relationship between the diverticula and cricopharyngeal muscles or between the diverticula, thyroid cartilage and cricoid cartilage.

## Background

Pharyngoesophageal diverticulum is a relatively rare disease and is classified into three types: Zenker’s diverticulum (ZD), Killian–Jamieson diverticulum (KJD), and Laimer’s diverticulum (LD). ZD is the most common form, followed by KJD and LD. Given its prevalence, cases of pharyngoesophageal diverticulum are often diagnosed as ZD. Before surgery, it is difficult to distinguish ZD and KJD.

We herein report a case of KJD diagnosed as ZD prior to surgery.

## Case presentation

**Fig. 1:**
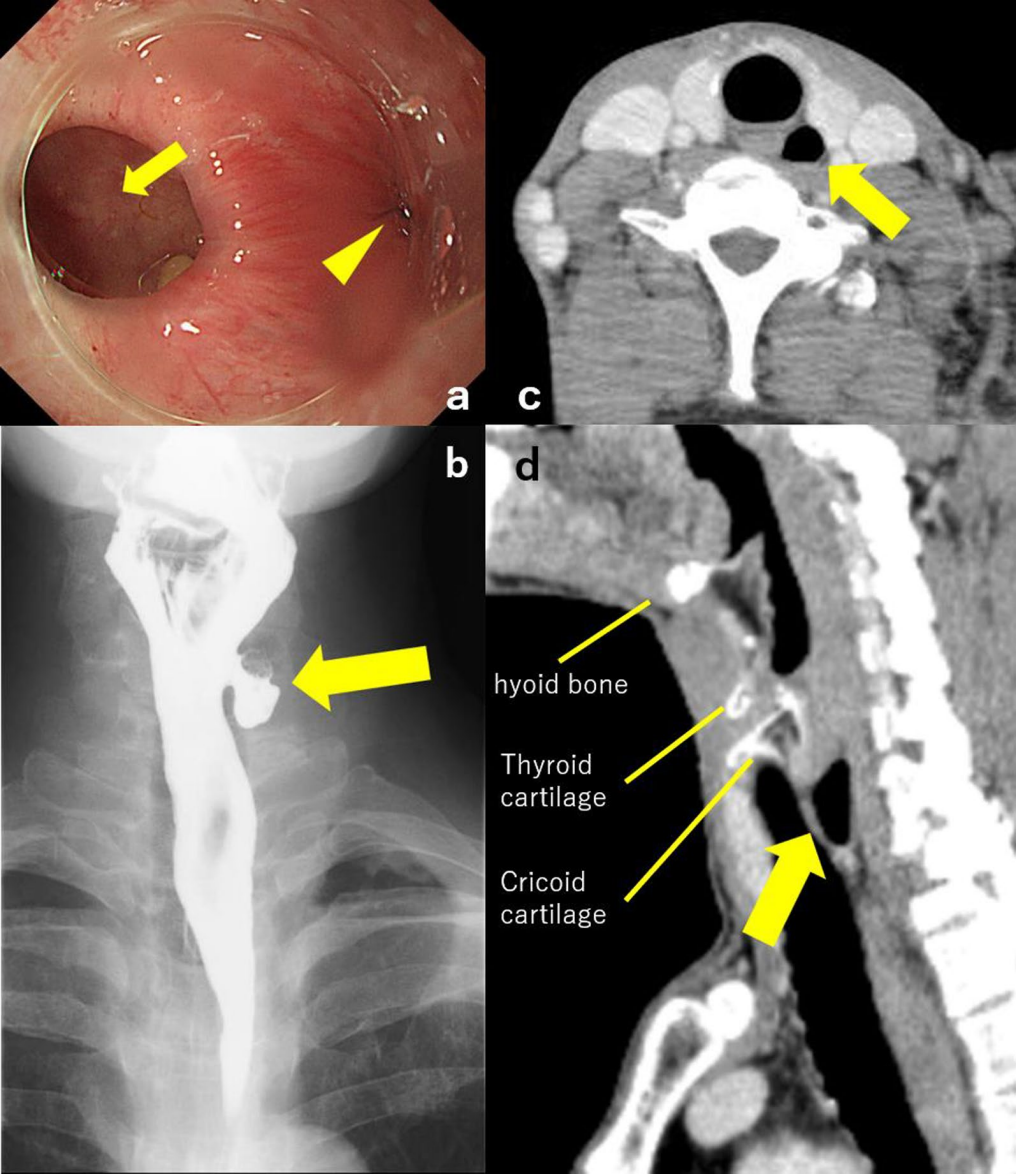
Preoperative findings. **a** Esophagogastroduodenoscopy showed a diverticulum (arrow) and the true lumen of the esophagus (arrowhead). **b** Esophagogram revealed the diverticulum at the left side of cervix (arrow). Axial (**c**) and sagittal (**d**) section of computed tomography showed an air-contained diverticulum arising from behind the left lobe of the thyroid (arrow). The diverticulum was diagnosed as Zenker’s diverticulum

A 56-year-old Japanese man was referred to our hospital with a 4-year history of discomfort during swallowing. His medical history was unremarkable. A physical examination revealed no abnormality. Esophagogastroduodenoscopy (EGD) showed a diverticulum filled with food residue just under the esophageal orifice (Fig. 1a). An esophagogram revealed an 18 × 10 × 12-mm diverticulum at the left side of the cervix (Fig. 1b). Contrast-enhanced cervical computed tomography (CT) showed an air-contained diverticulum arising from behind the left lobe of the thyroid (Fig. 1c, d). The diverticulum was diagnosed as ZD based on these endoscopic and radiographic findings.

**Fig. 2:**
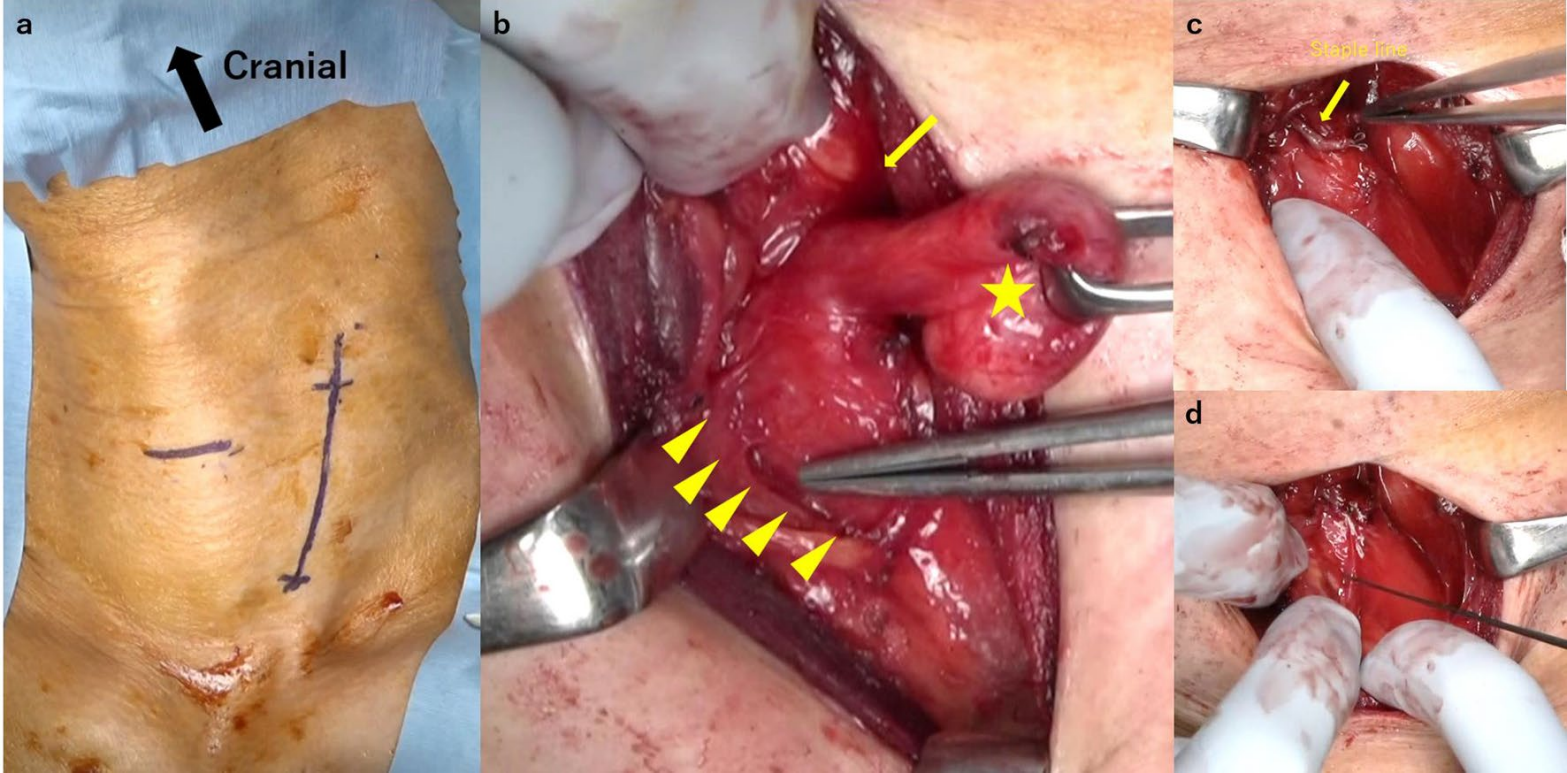
Intraoperative picture. **a** The cervical esophagus was accessed through a 6-cm left oblique incision on the anterior border of the left sternocleidomastoid muscle. **b** Diverticulum exposed to the base (star) and the cricopharyngeal muscle (arrow) and the left recurrent nerve (arrowheads). **c** The diverticulum was transected longitudinally using a linear stapler. **d** The left recurrent nerve was preserved, using intraoperative neuro monitoring

The patient underwent transcervical diverticulectomy under general anesthesia. He was placed in the supine position with neck extension. The cervical esophagus was accessed through a 6-cm left oblique incision on the anterior border of the left sternocleidomastoid muscle (Fig. 2a). The carotid artery was retracted laterally. The esophagus was detected at the medial side of the carotid artery, and the diverticulum was exposed by dissecting the layer of the prevertebral fascia. The diverticulum was carefully dissected toward the orifice and found to actually arise from the left wall of the esophagus. In detail, this diverticulum protruded from the level of the cricoid cartilage and below the cricopharyngeal muscle. This area is compatible with the Killian–Jamieson space, the muscular gap created between the esophageal longitudinal muscle and cricopharyngeal muscle (Fig. 2b). Therefore, we diagnosed the diverticulum as KJD during the operation.

The diverticulum was transected longitudinally using a linear stapler at the level of the orifice under endoscopy (Fig. 2c). We preserved the cricopharyngeal muscle and the left recurrent nerve and confirmed the function of the recurrent nerve via intra-operative neuro monitoring (IONM) (Fig. 2d). The staple line was buried and reinforced with absorbable suture. The histopathological diagnosis of the specimen was pseudodiverticulum without a muscular layer, compatible with KJD. No evidence of malignancy was found.

The postoperative course was uneventful, without vocal cord paralysis. Barium esophagography on postoperative day 3 confirmed that there was no leakage or remnant diverticulum. The patient was discharged on postoperative day 7 with a good food intake. At 2 months after surgery, he remains asymptomatic without recurrence of the diverticulum or stenosis at the surgical site.

## Discussion

Pharyngoesophageal diverticulum was first described by Ludlow in 1767 [1]. KJD was first reported by Ekberg and Nylander in 1983 [2]. The incidence of ZD, the most common type, has been reported as 2 per 100,000 capita per year [3, 4]. Furthermore, Ekberg and Wahlgren analyzed 854 patients with dysphagia by a pharyngoesophagographic examination; ZD was found in 20 patients (2.3%), and KJD was found in 16 patients (1.9%) [5].

**Fig. 3:**
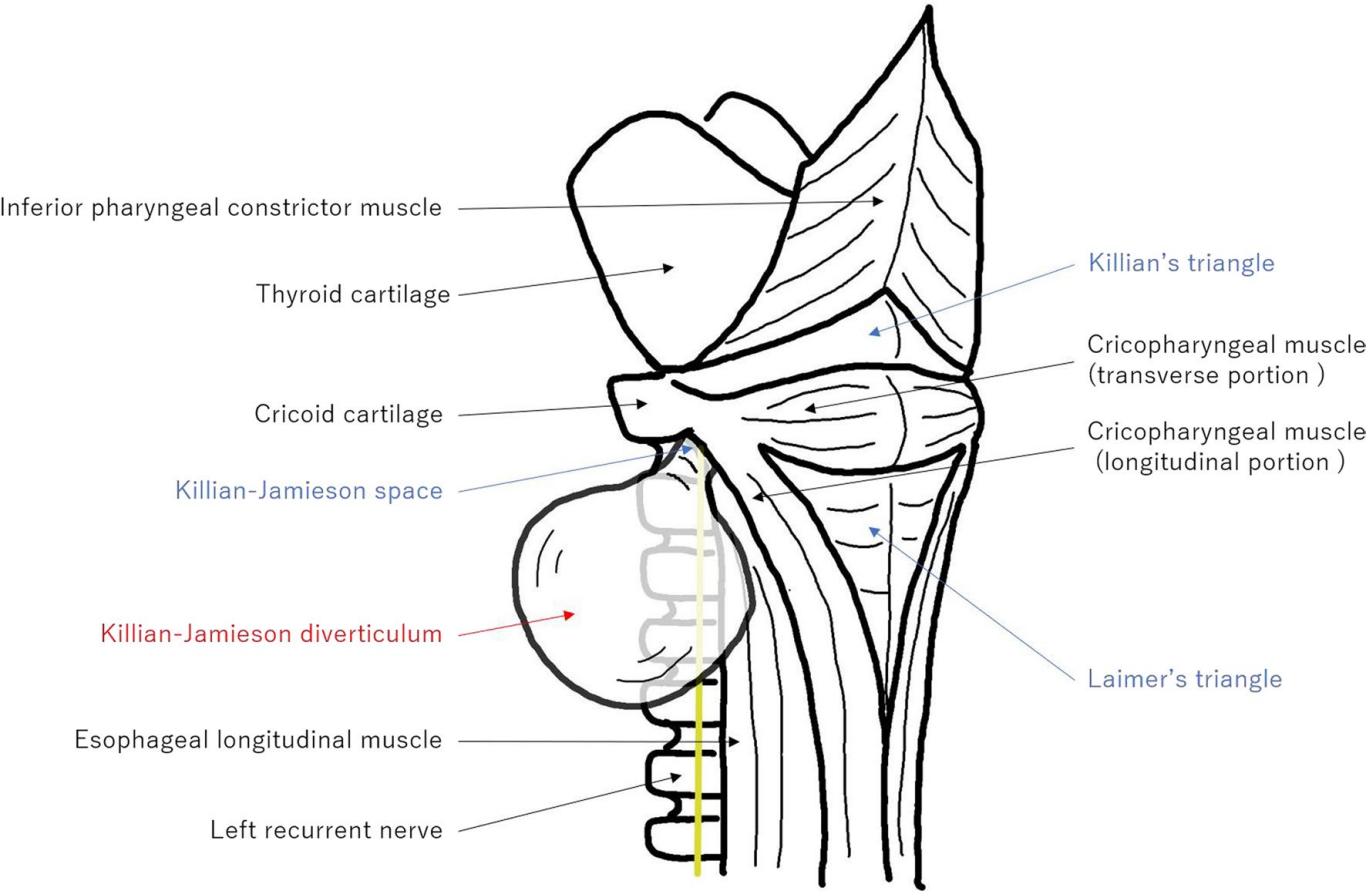
Anatomy of the hypopharynx and cervical esophagus

A pharyngoesophageal diverticulum is classified as ZD, KJD, or LD, each arising from different anatomically weak areas (Fig. 3). ZD arises from Killian’s triangle, a muscular gap in the posterior wall below the inferior pharyngeal constrictor muscle and above the cricopharyngeal muscle [6]. KJD arises from the Killian–Jamieson area, a triangle space just below the transverse portion of the cricopharyngeal muscle, superior and posterior to the esophageal longitudinal muscle, and superior and anterior to the longitudinal portion of the cricopharyngeal muscle [2]. LD arises from Laimer–Haeckerman’s triangle, an area covered only by the circular muscles of cervical esophagus on the dorsal side, between the esophageal longitudinal muscle and cricopharyngeal muscle [7, 8].

**Fig. 4:**
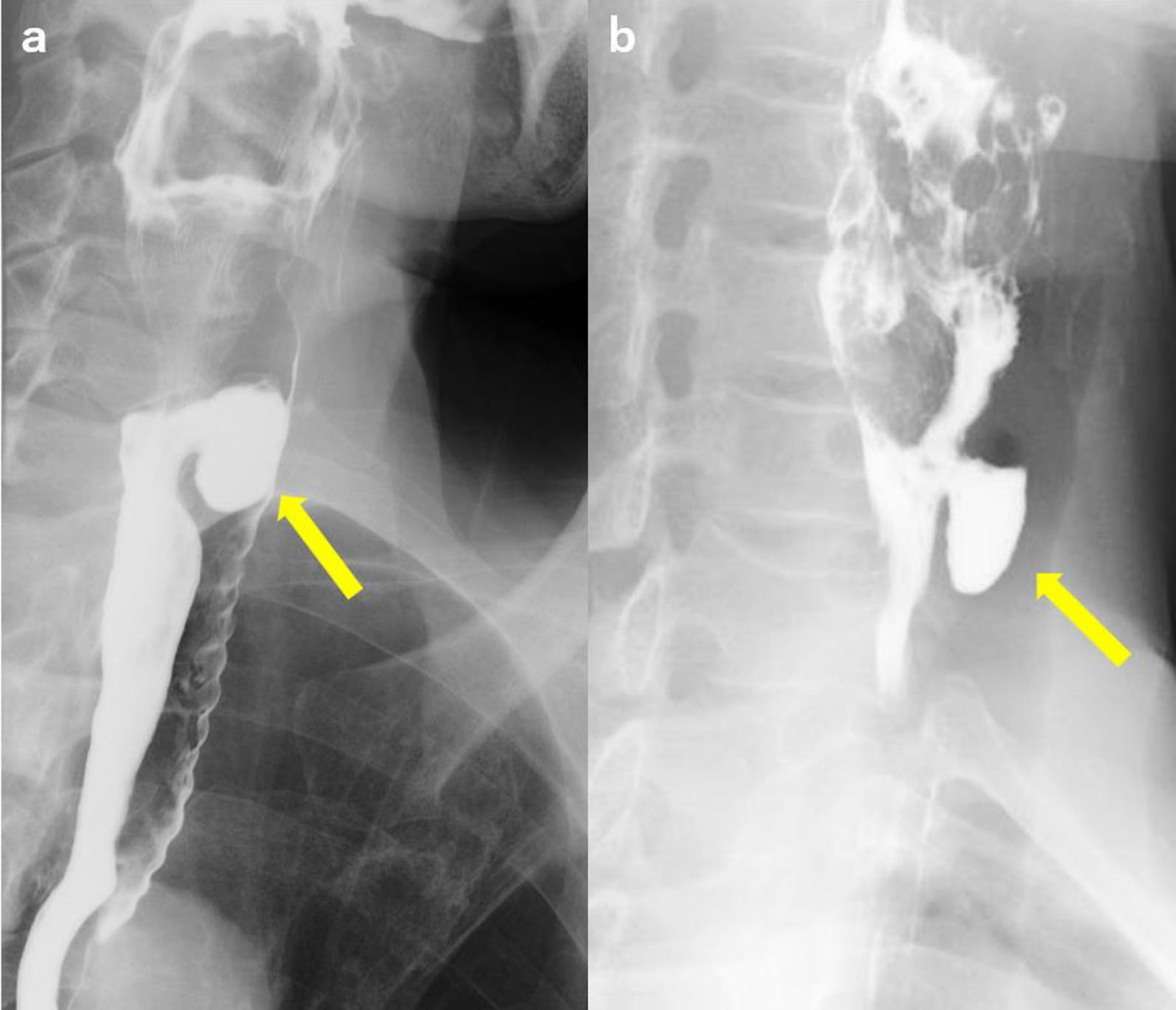
Comparison of esophagogram between Zenker diverticulum (arrow in **a**) and Killian–Jamieson diverticulum (arrow in **b**)

We diagnosed the present patient with pharyngoesophageal diverticulum by EGD and an esophagogram but could not distinguish ZD and KJD. Rubesin et al. distinguished ZD and KJD by an esophagogram [9]. The fluoroscopic image of esophageal motility in their study was obtained with the patient in the prone position. The cricopharyngeal muscle as the filling defect of cervical esophagus is a landmark for the diagnosis. The opening of ZD is above the defect, while the opening of KJD is below the defect. Considering this, we reviewed this KJD case and previous ZD cases in our hospital. However, diagnosing ZD or KJD based on radiological findings before surgery is difficult, as the barium defect of the cricopharyngeal muscle is difficult to detect in the clinical setting (Fig. 4).

**Fig. 5:**
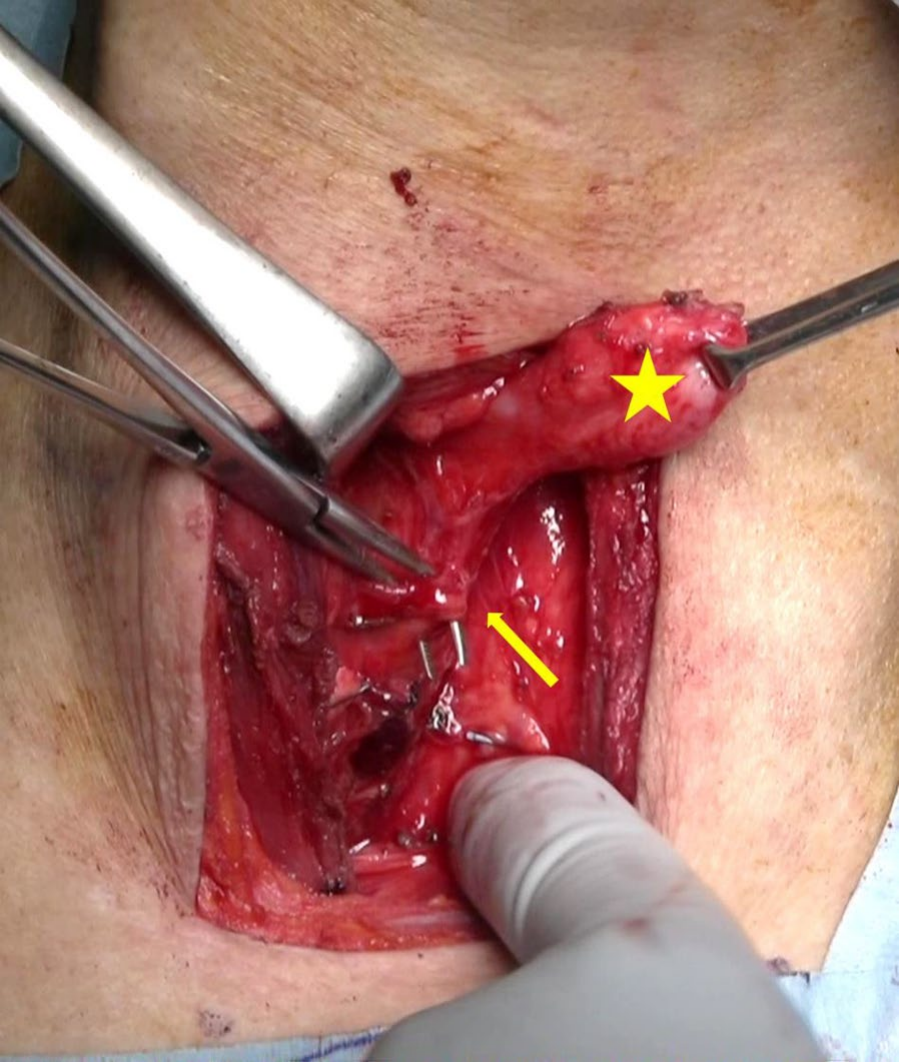
The case of Zenker diverticulum in our hospital. Intraoperative picture before resection of the diverticulum. The diverticulum (star) arose above the cricopharyngeal muscle (arrow)

In the present case, we diagnosed the pharyngoesophageal diverticulum as ZD before operation but found that the anatomy was actually KJD during the operation. In ZD cases, the diverticulum arises from the posterior wall of the esophagus and the cranial side of the cricopharyngeal muscle and cricoid cartilage, roughly at the level of the thyroid cartilage; however, in the present KJD case, the diverticulum arose from the left wall of the esophagus, at the level of the cricoid cartilage and below the cricopharyngeal muscle (Fig. 5). Such findings are usually difficult to detect based on CT images obtained before surgery and are also difficult to detect even after surgery with knowledge of the above-mentioned information, as the present case (Fig. 1c, d).

Distinguishing KJD and ZD is important because of two points: cricopharyngeal myotomy and preservation of recurrent nerve. In cases of ZD, cricopharyngeal myotomy is the gold standard for the prevention of recurrence [10]. On the other hand, in KJD cases, the cricopharyngeal myotomy (CPM) is controversial. Kim et al. reported that CPM should be performed to prevent the occurrence of ZD [11]. In contrast, Inoguchi et al. and Ebisumoto et al. reported that CPM increases the risk of gastroesophageal reflux among patients with swallowing difficulties [12, 13]. A previous report suggested that the cricopharyngeal muscle should be preserved in patients with KJD because of the risk of postoperative dysfunction of the muscle, which has the potential to cause issues such as gastroesophageal reflux [14]. CPM also has the potential to cause unnecessary esophageal injury. We first dissected the esophageal wall toward the caudal side and tried to cut the cricopharyngeal muscle, as we had considered this case to be ZD before surgery. Although the cricopharyngeal muscle was found on the cranial side of the diverticulum after a while (Fig. 2c), we performed an unnecessary procedure in this case. Fortunately, this dissection did not cause any unfavorable result, but there was still some risk of causing esophageal perforation or recurrent laryngeal nerve injury from this unnecessary procedure. Thus, recognizing cases of KJD is important for surgeons performing esophageal diverticulum operation, and a diagnosis should be made before surgery if possible. In addition, in KJD cases, the recurrent nerve is typically found immediately anterior to the orifice of the diverticulum. The Killian–Jamieson space is located at almost the same spot as the entrance of the recurrent nerve to the larynx. While the recurrent nerve may run near the orifice of the diverticulum even in ZD cases, in KJD cases, it is necessary to take particular care not to injure the recurrent nerve. In the present case, we confirmed that the function had been preserved by IONM.

Recently, endoscopic treatment has been reported in ZD and advantages, with respect to cosmesis and wound pain issues, have been reported [15–17]. On the other hand, there are some negative opinions about endoscopic treatment for KJD due to the problem of damage to the recurrent nerve and subsequent recurrence [11, 18]. In the near future, these issues might be overcome with increased experience and improvement of techniques [19]. However, at the present time, the open transcervical approach has advantages in distinguishing KJD from ZD, and in avoiding the recurrent laryngeal nerve injury by visualization and the use of IOMN. Indeed, the open transcervical approach was selected in the present case, and the patient could be discharged without any trouble or subsequent recurrence.

## Conclusion

We experienced a case of KJD, a relatively rare type of pharyngoesophageal diverticula. When treating pharyngoesophageal diverticula, surgeons should be aware of this type of diverticulum because the need to perform cricopharyngeal myotomy and the course of the RLN differ from cases of ZD. To make an accurate diagnosis, clinical and surgical findings are important, including the location of the diverticulum and the relationship between the diverticula and cricopharyngeal muscles or between the diverticula, thyroid cartilage, and cricoid cartilage.

## Data Availability

This data is available at Tohoku University Hospital.

## Abbreviations

KJD: Killian–Jamieson diverticulum
ZD: Zenker’s diverticulum
LD: Laimer’s diverticulum
CT: Computed tomography
IONM: Intra-operative neuro monitoring
CPM: Cricopharyngeal myotomy
